# Hematoma-derived exosomes of chronic subdural hematoma promote abnormal angiogenesis and inhibit hematoma absorption through miR-144-5p

**DOI:** 10.18632/aging.102550

**Published:** 2019-12-16

**Authors:** Chuang Gao, Zhitao Gong, Dong Wang, Jinhao Huang, Yu Qian, Meng Nie, Weiwei Jiang, Xuanhui Liu, Hongliang Luo, Jiangyuan Yuan, Tangtang Xiang, Shuo An, Wei Quan, Huijie Wei, Jianning Zhang, Rongcai Jiang

**Affiliations:** 1 Department of Neurosurgery, Tianjin Medical University General Hospital, Tianjin, China; 2 Tianjin Neurological Institute, Key Laboratory of Post-Neuroinjury Neuro-repair and Regeneration in Central Nervous System, Ministry of Education, Tianjin, China

**Keywords:** chronic subdural hematoma, exosomes, miR-144-5p, angiogenesis, hematoma absorption

## Abstract

Exosomes are small (30–150 nm diameter) lipid bilayer-enclosed vesicles found in all bodily fluids. We investigated whether exosomes play a role in chronic subdural hematoma (CSDH). Exosomes were identified and characterized using transmission electron microscopy and NanoSight particle tracking. The functions of hematoma-derived exosomes were evaluated in a rat model of acute subdural hematoma (SDH). The hematoma-derived exosomes inhibited hematoma absorption and exacerbated neurological deficits in SDH rats. We examined the effects of the exosomes on angiogenesis and cell permeability in human umbilical vein endothelial cells (HUVECs). Co-culture of exosomes with HUVECs revealed that the hematoma-derived exosomes were taken-in by the HUVECs, resulting in enhanced tube formation and vascular permeability. Additionally, there was a concomitant increase in ANG-2 expression and decrease in ANG-1 expression. Exosomes were enriched with microRNAs including miR-144-5p, which they could deliver to HUVECs to promote angiogenesis and increase membrane permeability. Overexpression of miR-144-5p in HUVECs and in SDH rats promoted abnormal angiogenesis and reduced hematoma absorption, which mimicked the effects of the hematoma-derived exosomes both *in vitro* and *in vivo*. Thus, hematoma-derived exosomes promote abnormal angiogenesis with high permeability and inhibit hematoma absorption through miR-144-5p in CSDH.

## INTRODUCTION

The incidence of chronic subdural hematoma (CSDH) is increasing in the general population, particularly among elderly individuals [1]. It is approximately 1–13/100,000 in the general population [2] and 129.5/100,000 among individuals 80 years of age and older [3]. Given that the number of individuals over 65 years of age will more than triple by 2030, CSDH is projected to become the most common cranial neurosurgical condition in the United States [4]. Although most CSDH patients achieve satisfactory outcomes following surgery, the rate of recurrence ranges from 2.5–33% [5]. Additionally, long-term outcomes among elderly patients are poor (mortality rates of 26.3% and 32% at 6 months and 1 year post-surgery, respectively) [6]. The major causes of death are disturbance of consciousness at onset and pneumonia [7].

Dexamethasone, atorvastatin, tranexamic acid, angiotensin-converting enzyme inhibitors, and other locally acting drugs have been investigated for the treatment of CSDH [8]. Atorvastatin is the only drug that is supported by class I evidence. However, a previous study showed that 11.2% of patients treated with atorvastatin had no response to the therapy and required surgical intervention. Additionally, therapeutic efficacy was frequently not achieved for 4 weeks or longer [2]. Thus, an understanding of the pathophysiologic mechanisms underlying CSDH is important in order to develop more effective, non-surgical treatments for the condition [9].

Angiogenesis and a chronic inflammatory reaction may play a role in CSDH formation and enlargement [10]. The outer subdural membrane of the hematoma contains immature blood vessels that are highly permeable and contain many small vesicles [11]. Inflammatory cells including neutrophils, lymphocytes, monocytes, and eosinophils have also been observed on the outer membrane [12]. Inflammatory and pro-angiogenic cytokines including TNF-a, IL-6, IL-8, VEGF, MMP-2, and MMP-9 are present at high concentrations in hematomas and have been correlated with recurrence [10, 13]. The high concentrations of soluble cytokines in CSDH fluid are thought to originate from the outer subdural membrane [14, 15]. However, it is not clear how the expression of these cytokines is regulated.

Exosomes are endosome-derived, membrane-bound vesicles with diameters of approximately 30–150 nm [16]. They are found in serum, urine, cerebrospinal, and other bodily fluids where they regulate intercellular communication [17]. MicroRNAs (miRNAs) regulate gene expression at the post-transcriptional level and are involved in processes such as cell proliferation and migration, stem cell differentiation, inflammation, and apoptosis [18]. Exosomes can transfer miRNAs to endothelial cells to promote vascular permeability and angiogenesis [19, 20]. Additionally, the levels of exosomal miRNAs and proteins may function as biomarkers for diagnosis and treatment response in various neurological disorders including traumatic brain injury, stroke, and neurodegenerative diseases [21–23].

Hematoma fluid primarily originates from blood [24]. In this study, we investigated whether exosomes are present in hematoma fluid and play a role in the pathogenesis of CSDH. We examined the effects of hematoma-derived exosomes on angiogenesis and vascular permeability in human umbilical vein endothelial cells (HUVECs) *in vitro*. Additionally, we evaluated the effects of the exosomes on angiogenesis, hematoma absorption, and cognitive function in a rat model of acute subdural hematoma (SDH) *in vivo*. Finally, miRNA sequencing and up-regulation of mir-144-5p was performed to study the underlying mechanisms.

## RESULTS

### Identification of hematoma-derived exosomes

**Figure 1:**
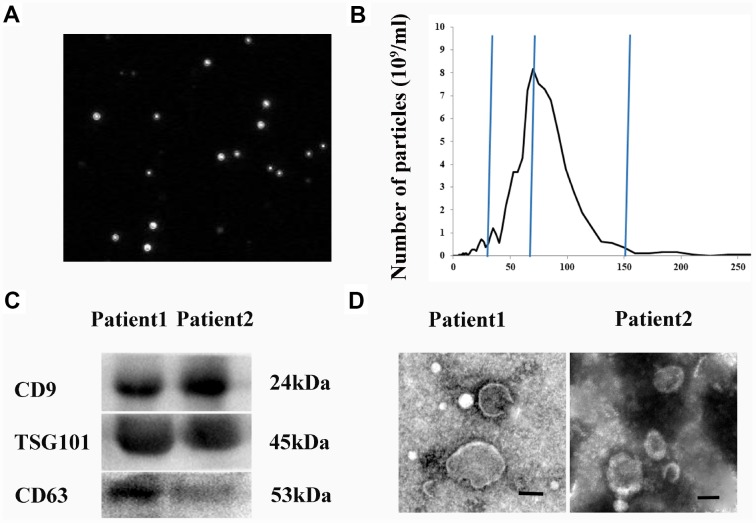
**Isolation and characterization of hematoma-derived exosomes.** (**A**) Representative images from NanoSight particle tracking analysis. (**B**) Size distribution of hematoma-derived exosomes. (**C**) Expression of CD9, CD63, and TSG101 in hematoma-derived exosomes. (**D**) Representative TEM image of exosomes. Scale bar: 100 nm.

We isolated and characterized exosomes derived from hematomas from CSDH patients. NanoSight particle tracking analysis was performed to evaluate exosome size and particle number (Figure 1A). The diameters of the exosomes ranged from 30–150 nm (peak at 74 nm) (Figure 1B). Exosomes expressed typical exosomal markers including CD9, CD63, and TSG101 (Figure 1C). Finally, exosome morphology was evaluated using transmission electron microscopy (TEM) (Figure 1D).

### Hematoma-derived exosomes inhibit hematoma absorption and exacerbate neurological deficits *in vivo*

**Figure 2:**
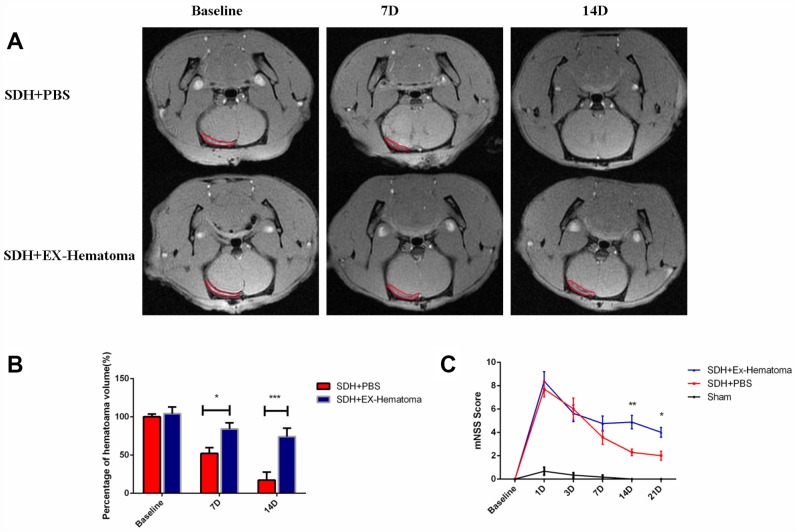
**The effects of hematoma-derived exosomes on hematoma absorption and neurological function.** (**A**) Representative MR images of SDH rats at baseline, and 7 and 14 days after injection. (**B**) Quantification of hematoma volume. No differences in hematoma volume were observed at baseline, while the EX-Hematoma group had higher hematoma volumes on days 7 and 14 compared to the PBS control group. (**C**) The EX-Hematoma group had higher mNSS on days 14 and 21. Values are shown as the mean ± SEM, Two-way ANOVA, *p < 0.05, ** p < 0.01, *** p < 0.001.

We evaluated the effects of hematoma-derived exosomes on hematoma absorption and neurological function in a rat model of acute SDH. Magnetic resonance imaging (MRI) was performed to assess hematoma volume at baseline and at 7, 14, and 21 days after injection of the exosomes (Figure 2A). No differences in hematoma volume were observed among rats treated with hematoma-derived exosomes (EX-Hematoma group) compared to controls (PBS group) at baseline (Figure 2B). However, higher hematoma volumes were observed in the EX-Hematoma group compared to controls 7 and 14 days after injection (Figure 2B). We evaluated neurological deficits using the Modified Neurological Severity Score (mNSS). The mNSS scores were higher in SDH rats compared to controls (sham), consistent with previous studies [25]. We found that the mNSS scores were higher in the EX-Hematoma group compared to controls 14 and 21 days after injection (Figure 2C).

### Hematoma-derived exosomes prompts abnormal angiogenesis with increased vascular permeability *in vitro*

**Figure 3:**
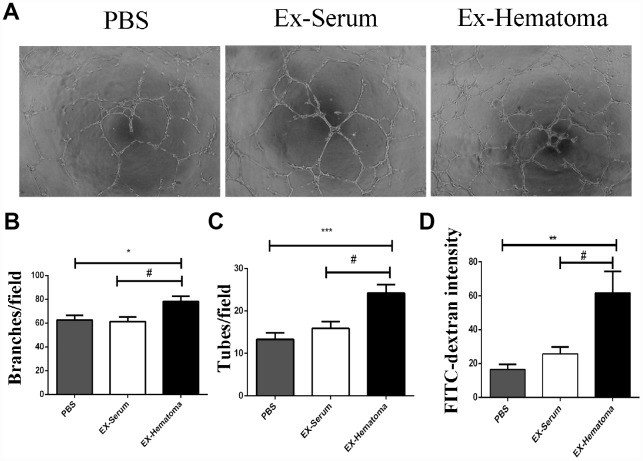
**The effects of hematoma-derived exosomes on tube formation and vascular permeability in HUVECs.** (**A**) Representative images of tube formation. (**B**, **C**) Quantification of branch and tube formation. (**D**) Permeability of HUVEC monolayers to FITC-Dextran. The FITC-Dextran intensity was higher in the EX-Hematoma compared to the EX-Serum group. * p < 0.05, ** p < 0.01, *** p < 0.001, # p < 0.05.

Angiogenesis plays an important role in the pathogenesis of SDH [25]. We therefore investigated the effects of hematoma-derived exosomes on angiogenesis in HUVECs using *in vitro* tube formation assays. We observed an increase in branches and tubes in HUVECs treated with hematoma-derived exosomes (EX-Hematoma group) compared to those treated with serum-derived exosomes (EX-serum group) and those treated with PBS (control group) (Figure 3A–3C). We also investigated the effects of the exosomes on the permeability of HUVEC monolayers *in vitro*. The intensity of the FITC-Dextran was higher in the EX-Hematoma compared to EX-Serum group, indicating hematoma-derived exosomes increased vascular permeability (Figure 3D).

**Figure 4:**
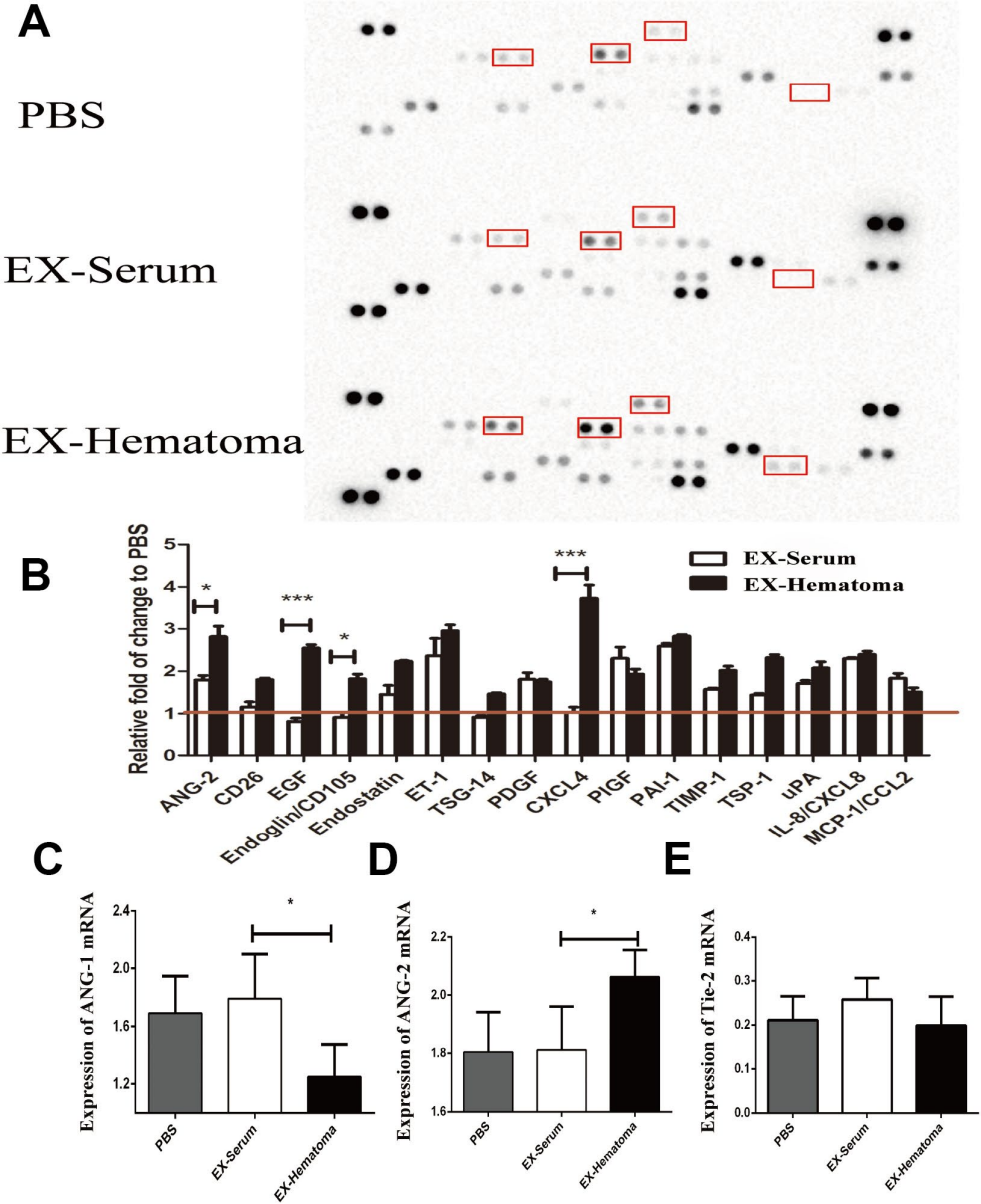
**The effects of hematoma-derived exosomes on angiogenic cytokine expression.** (**A**) Representative images of the cytokine array. (**B**) Quantification of cytokine expression. ANG-2, EGF, Endoglin, and CXCL4 expression were higher in the EX-Hematoma compared to the EX-Serum group. (**C**–**E**) RT-PCR analysis of ANG-1, ANG-2, and Tie-2 mRNA expression following co-culture with exosomes. ANG-2 mRNA expression was higher, while ANG-1 expression was lower in the EX-Hematoma compared to the EX-Serum group. * p < 0.05, *** p < 0.001.

We next investigated the effects of the exosomes on the expression of angiogenesis-related cytokines. Hematoma-derived exosomes were co-cultured with HUVECs for 24 hrs and the expression of angiogenesis-related cytokines was quantified using cytokine arrays. ANG-2, EGF, Endoglin, and CXCL4 expression were higher in HUVECs treated with hematoma-derived exosomes (EX-Hematoma group) compared to those treated with serum-derived exosomes (EX-Serum group) (Figure 4B). Interestingly, ANG-2 mRNA expression was higher while ANG-1 expression was lower in the EX-Hematoma compared to EX-Serum group (Figure 4C and 4D). However, no differences in Tie-2 (angiopoietin receptor) mRNA expression were observed between groups (Figure 4E).

### ANG-1 and ANG-2 expression in hematomas and serum of CSDH patients

**Figure 5:**
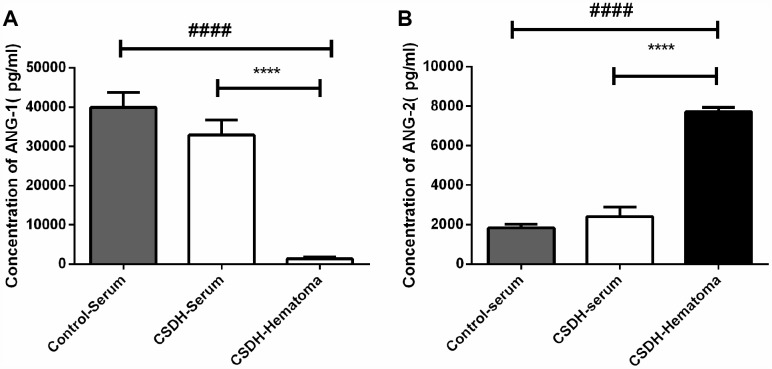
**Analysis of ANG-1 and ANG-2 concentrations in serum from CSDH patients, serum from healthy controls, or hematoma supernatants.** (**A**) No differences in the serum ANG-1 concentration were observed between CSDH patients and healthy controls. The ANG-1 concentration was lower in hematoma supernatants compared to the concentration in serum. (**B**) No differences in the ANG-2 concentration were observed in serum from CSDH patients and healthy controls. The ANG-1 concentration was higher in hematoma supernatants compared to in serum. Data are presented as the mean ± SEM. **** p < 0.0001, #### p < 0.0001.

The ANG-1/ANG-2 mRNA expression was previously demonstrated to be lower in the neo-membranes of CSDH compared to the dura (0.48 vs. 1.9, respectively) [15]. We investigated the concentrations of ANG-1 and ANG-2 in hematoma and serum samples from CSDH and healthy control patients. No differences in clinical parameters including age, gender, and laboratory values (e.g. routine blood, liver function, and renal function tests) were observed between groups (Supplementary Table 2). The concentrations of ANG-1 and ANG-2 were comparable in serum samples from CDSH and healthy controls. However, the concentration of ANG-1 was lower, while the concentration of ANG-2 was higher in hematoma compared to serum samples from CDSH patients (Figure 5A, 5B).

### Hematoma-derived exosomes deliver miR-144-5p to HUVECs

**Figure 6:**
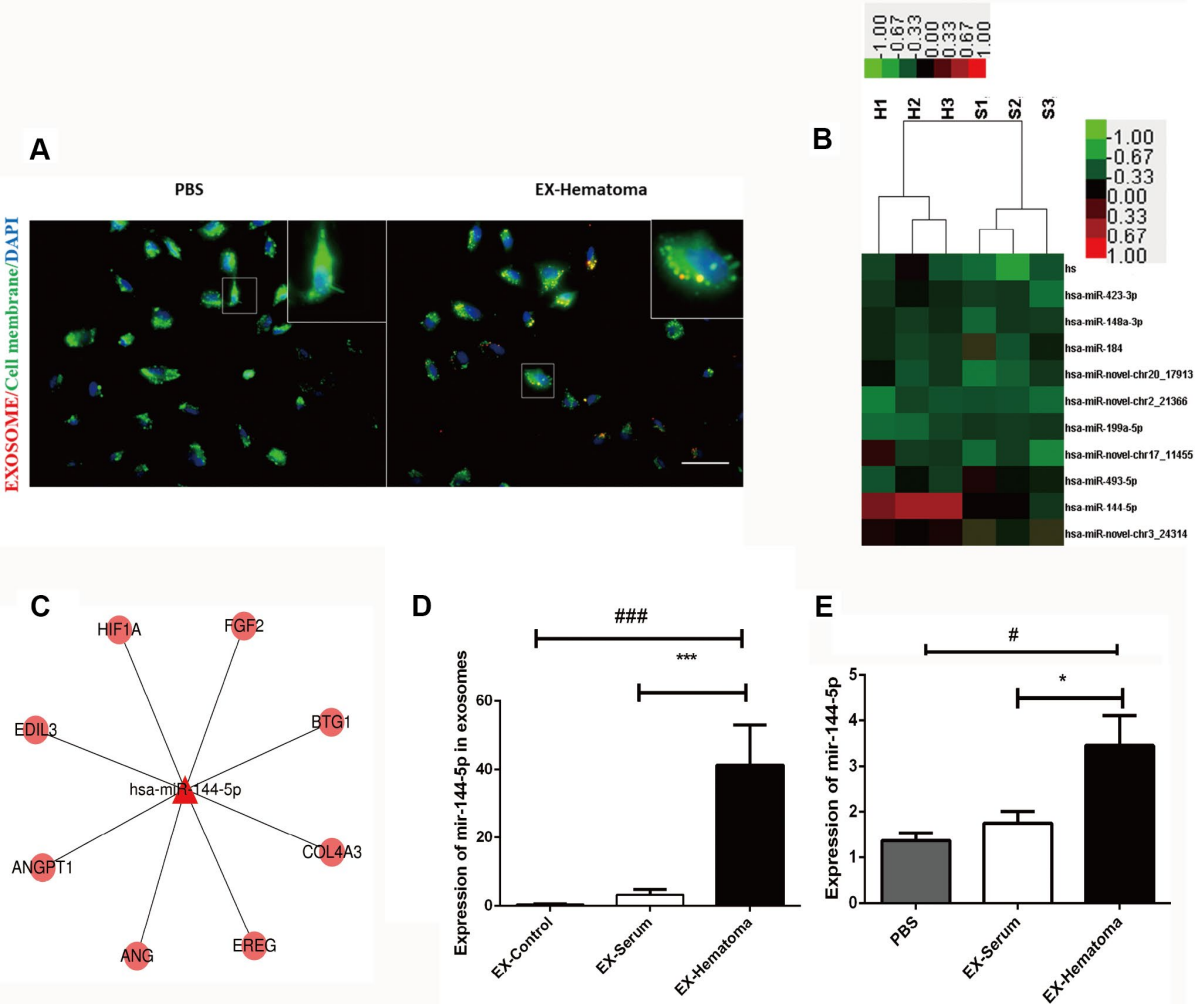
**Hematoma-derived exosomes are enriched with miR-144-5p, which they can transfer to HUVECs.** (**A**) PKH26-stained hematoma-derived exosomes (red) were internalized by HUVECs (green; scale bar: 50 μm). (**B**) Heat map showing hierarchical clustering of altered miRNAs. Values represent the log2 (fold change) in miRNA expression relative to the expression in serum-derived exosomes from healthy controls. (**C**) Network of miR-144-5p. (**D**) RT-PCR confirming expression of miR-144-5p in hematoma-derived exosomes. (**E**) Quantitative analysis of miR-144-5p in HUVECs following co-culture with exosomes. MiR-144-5p expression was higher in the EX-Hematoma compared to EX-Serum group. * p < 0.05, # p < 0.05, *** p < 0.001, ### p < 0.001.

We investigated whether HUVECs could internalize hematoma-derived exosomes. Purified hematoma-derived exosomes co-localized with HUVECs following co-culture, suggesting that exosomes could be taken up by HUVECs (Figure 6A). Given that miRNAs are abundant in exosomes [26], we hypothesized that exosome-derived miRNAs could regulate gene expression in HUVECs. We therefore performed miRNA sequencing to evaluate differences in miRNAs content between hematoma- and serum-derived exosomes. The sequencing data were shown in Supplementary Tables 3 and 4. The miRNA expression patterns were similar among all types of exosomes (Supplementary Figure 1). Alterations in seven miRNAs were observed in hematoma- compared to serum-derived exosomes. Five miRNAs were upregulated while two were downregulated (two-fold change, adjusted P < 0.05) (Figure 6B). Of these miRNAs, miR-144-5p exhibited the largest difference in expression. We confirmed that miR-144-5p expression was higher in hematoma- compared to serum-derived exosomes by RT-PCR (Figure 6D). Thus, uptake of exosomes by HUVECs results in an increase in miR-144-5p expression (Figure 6E).

### MiR-144-5p overexpression promotes angiogenesis and reduces hematoma absorption *in vitro* and *in vivo*

**Figure 7:**
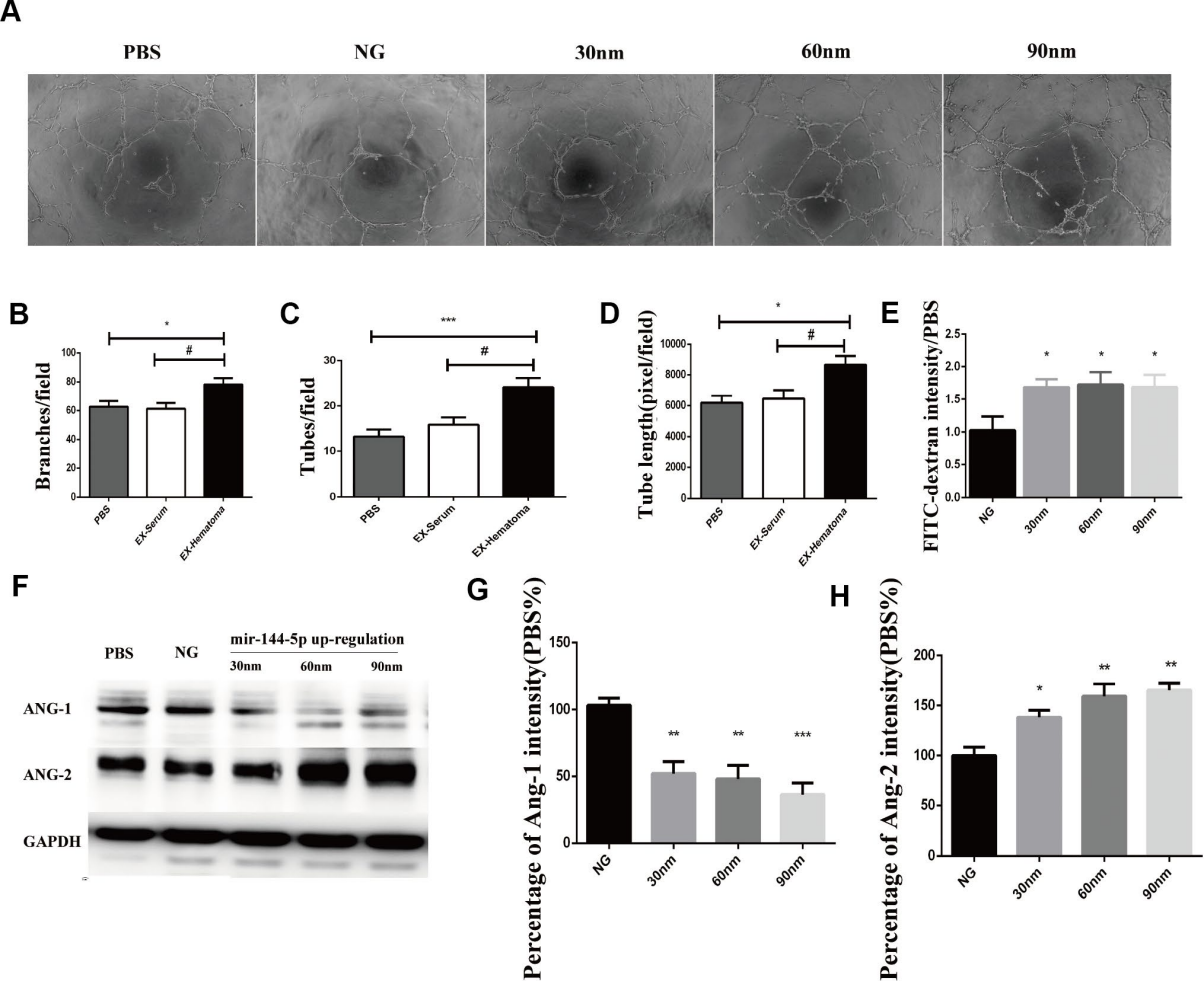
**Over-expression of miR-144-5p promoted tube formation, increased cell permeability, increased ANG-2 expression, and decreased ANG-1 expression in HUVECs.** (**A**) Representative images of tube formation. (**B**–**D**) Quantification of branches, tubes, and tube length. (**E**) Effects of miR-144-5p over-expression on the permeability of HUVEC monolayers to FITC-Dextran. (**F**) Representative images of western blots showing ANG-1 and ANG-2 expression. (**G**–**H**) Quantification of ANG-1 and ANG-2 expression.* p < 0.05, ** p < 0.01, # p < 0.05.

**Figure 8:**
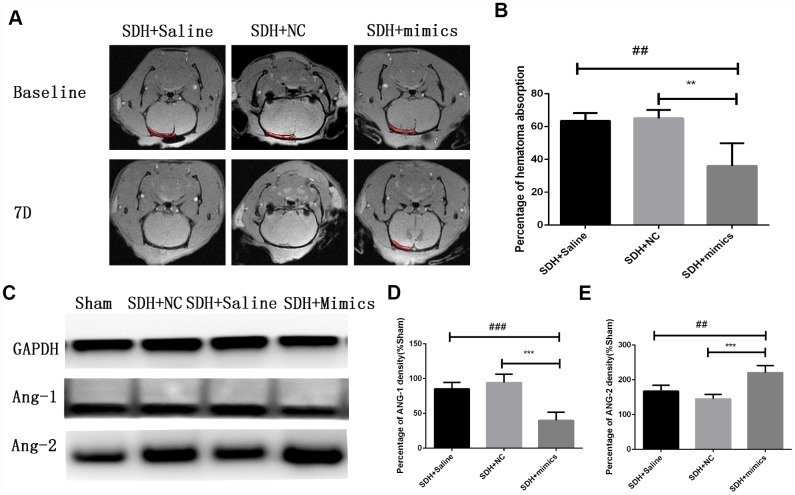
**Over-expression of miR-144-5p results in decreased hematoma absorption, increased ANG-2 expression, and decreased ANG-1 expression in SDH rats.** (**A**) Representative MR images of SDH rats at baseline and day 7. (**B**) Quantification of the percentage of hematoma absorption at baseline compared to day 7. Decreased hematoma absorption on day 7 was observed in the SDH + miRNA mimic group compared to the saline and negative control (NC) groups. (**C**) Representative images of western blots demonstrating differences in ANG-1 and ANG-2 expression. (**D,** **E**) Quantification of ANG-1 and ANG-2 expression. ** p < 0.01, ***p < 0.001, ## p < 0.01, ### p < 0.001.

We overexpressed miR-144-5p in HUVECs Supplementary Figure 2 and analyzed the effects on angiogenesis *in vitro*. Transfection of HUVECs with miR-144-5p resulted in an increase in tube number, branch formation, and tube length (Figure 7A–7D). Additionally, miR-144-5p over-expression resulted in increased permeability of HUVEC monolayers to FITC-Dextran (Figure 7E). Western blot analysis revealed that miR-144-5p over-expression resulted in increased ANG-2 and decreased ANG-1 expression in HUVECs (Figure 7F–7H). Finally, we investigated the effects of miR-144-5p over-expression in SHD rats. Over-expression resulted in an increase in miR-144-5p on hematoma membranes (Supplementary Figure 3) and reduced hematoma absorption (Figure 8A, 8B). Increased ANG-2 and decreased ANG-1 were observed 7 days after induction were observed 7 days after induction, consistent with the results of the *in vitro* studies (Figure 8C–8E).

## DISCUSSION

CSDH is a cerebrovascular disease mediated by chronic inflammation and angiogenesis [27]. Numerous newly formed capillaries with enlarged blood vessels and a lack of a basement membrane have been observed in the outer membrane of the hematoma. The newly formed capillaries tear easily and are highly permeable, which can result in re-bleeding and exudation [28]. Hemorrhage accounts for 0.2–28.6% of hematoma content, suggesting that continuous or intermittent hemorrhage may play an important role in CDSH formation and progression [29].

We found that exosomes can promote angiogenesis with an increased cell permeability and expression of ANG-2, which indicated the newly formed capillaries were immature. Additionally, we showed that hematoma-derived exosomes reduced hematoma absorption, which could be a consequence of exosome-induced abnormal angiogenesis. Previous proteomic analysis demonstrated highly similar expression patterns in CSDH fluid and serum [24]. We found that miRNA expression was similar in hematoma- compared to serum-derived exosomes. However, higher miR-144-5p expression was observed in hematoma-derived exosomes compared to serum-derived exosomes. Several studies have shown that miR-144-5p is a prognostic biomarker in esophageal [30], gastric [31], and breast cancer [32]. High miR-144-5p expression in chronic periodontitis was negatively correlated with COX2 and IL17F expression, suggesting that miR-144-5p could play a role in chronic inflammation [33]. Finally, miR-144-5p was shown to inhibit SDC3 expression [34], which could suppress angiogenesis by inhibiting endothelial cell migration and tube formation [35]. We found that co-culture of HUVECs with hematoma-derived exosomes resulted in an increase in miR-144-5p in HUVECs and promoted angiogenesis and cell permeability by altering ANG-1 and ANG-2 expression. Thus, targeting miR-144-5p may be a potential therapeutic strategy for CSDH.

ANG-1 and ANG-2 have important roles in angiogenesis. ANG-1 regulates blood vessel maturation and endothelial cell adhesion and migration. Mice that overexpress Ang1 in the skin have larger and more highly branched vessels that are resistant to leakage induced by inflammatory stimuli [36, 37]. ANG-2 is primarily secreted by endothelial cells at sites of active vascular remodeling where it promotes dissociation of pericytes from pre-existing vessels and increases vascular permeability [38]. ANG-1 expression is higher in mature vascular beds compared to ANG-2, which promotes blood vessel maturation, microvascular network stabilization, and reduced vascular permeability. Our data indicate that ANG-2 expression in higher while ANG-1 expression is lower in hematomas compared to serum. Since we observed no differences in the ANG-1 and ANG-2 concentrations in serum from CSDH patients compared to healthy controls, we hypothesize that the higher concentrations of ANG-2 result from local synthesis rather than migration from the peripheral blood.

In summary, we have demonstrated that exosomes are present in the hematomas of CSDH patients. Hematoma-derived exosomes are enriched with miR-144-5p, which they deliver to HUVECs resulting in increased abnormal angiogenesis and vascular permeability, and decreased hematoma absorption. Thus, angiogenesis and blood vessel maturation should be considered when attempting to treat CSDH non-surgically. Hematoma-derived exosomes and miR-144-5p are potential therapeutic targets in CSDH.

## METHODS

### Hematoma and serum sample collection

Twenty CSDH and 10 matched healthy control patients were enrolled in the study between 2016 and 2017. All patients provided written informed consent. Peripheral blood samples were collected upon admission to the Neurosurgical Department of Tianjin Medical University General Hospital. Hematoma samples were collected from patients who underwent burr hole craniotomy. Blood and hematoma samples were centrifuged at 2000 × g for 20 min at 4°C. Serum and hematoma supernatants were then aliquoted and stored at −80°C until use.

### Exosome isolation and characterization

Exosomes derived from serum or hematomas were extracted by differential ultracentrifugation as described [39]. Debris was removed by centrifugation at 2000 x g for 20 min at 4°C. The samples were then centrifuged at 10,000 x g for 30 min at 4°C. The supernatants were collected and the samples centrifuged at 100,000 x g for 2 hrs. The resulting pellets were resuspended in PBS, passed through a 0.22 μm filter, and then centrifuged at 100,000 x g for 70 min. Finally, the precipitates were resuspended in either PBS for NanoSight particle tracking analysis, Trizol for RNA extraction, or radioimmunoprecipitation assay (RIPA) buffer for western blotting.

### NanoSight particle tracking analysis

Exosomes (1 × 10^7^ / mL to 1 × 10^9^ / mL) were visualized using a NanoSight NS300 equipped with a 405 nm laser (Malvern, Great Malvern, UK). Exome size and number of particles were assessed. Videos (60 s duration, 30 frames / sec) were recorded and particle movement analyzed using the NTA software (NanoSight version 2.3).

### Western blotting

Hematoma samples were ultracentrifuged and the precipitates lysed on ice in ice-cold RIPA buffer for 15 min. Lysates were then centrifuged at 13,000 x g for 10 min. Exosome concentrations were estimated using bicinchoninic acid (BCA) assays (BCA1-1KT, Sigma Aldrich, USA). The expression of exosome markers on hematoma-derived vesicles was evaluated using the following antibodies: mouse anti-CD63 (ab59479, Abcam, Cambridge, UK, 1:500 dilution), mouse anti-TSG101 (ab125011, Cambridge, UK, Abcam, 1:500), and mouse anti-CD9 (ab92726, Cambridge, UK, Abcam, 1:500). The concentrations of ANG-1 and ANG-2 in HUVECs were evaluated by western blotting using rabbit anti-ANG-1 (ab8451, Abcam, Cambridge, UK, 1:500), rabbit anti-ANG-2 (ab8452, Abcam, Cambridge, UK, 1:500) antibodies, and rabbit anti-GAPDH as a loading control (ab181602, Abcam, Cambridge, UK, 1:500).

### TEM

Exosomes were fixed in 2% paraformaldehyde and adsorbed onto formvar/carbon-coated 200-mesh nickel grids (Electron Microscopy Sciences) for 15 min. The grids were then washed with PBS, fixed in 2.5% glutaraldehyde for 5 min, and rinsed with milliQ water. Finally, the grids were negatively stained with 2% uranyl acetate for 1 min and then washed and air-dried overnight. Images were acquired on a Zeiss EM900 microscope (Carl Zeiss Microscopy GmbH) equipped with a wide-angle, dual-speed 2K-CCD camera at 80 kV.

### MiRNA sequencing

Exosomes were resuspended in 500 uL of Trizol (Invitrogen, USA) for high-throughput miRNA sequencing (Cloud-Seq Biotech, Shanghai, China). Briefly, total RNA was extracted and the A260/A280 ratio measured using a NanoDrop ND-1000 (Thermo Fisher Scientific, Waltham, MA, USA). All samples had an A260/A280 ratio > 1.8. The miRNA sequencing library was established and the quality assessed using an Agilent 2100 Bioanalyzer (Agilent Technologies, Santa Clara, CA). The library was denatured and the single-stranded DNA molecules captured on Illumina flow cells. DNA was then amplified *in situ* as clusters and amplified for 50 cycles on an Illumina HiSeq sequencer according to the manufacturer's instructions. All results were confirmed by RT-PCR.

### HUVEC and exosome co-culture

HUVECs (ATCC, USA) were cultured in Endothelial Growth Medium-2 (EGM-2, Lonza, USA) supplemented with 10% exosome-depleted Fetal Bovine Serum (Gibco, Cat. A2720801, USA). The media was replaced every 2 days. Exosomes isolated from either 1 mL serum or hematoma supernatants were ultracentrifuged, passed through a 0.22 μm filter, dissolved in 1 mL culture medium, and then incubated with HUVECs for 24 hrs at 37°C in a 5% CO_2_ incubator.

### RT-PCR

HUVECs or exosomes were lysed in Trizol (Invitrogen, USA) and the resulting lysates mixed with chloroform and centrifuged at 13,000 x g for 15 min at 4°C. The aqueous phase (top layer) was collected and mixed with isopropanol to precipitate total RNA. The cDNA was synthesized with 1 μg of total RNA using the SuperScript™ IV VILO™ Master Mix kit according to the manufacturer’s protocol (Thermo Fisher Scientific, Cat. 11756500). Real-time PCR was performed with the PowerUp™ SYBR™ Green kit (Thermo Fisher Scientific, Cat. A25742) according to the manufacturer's instructions. RNA expression was normalized to GAPDH. Relative gene expression was analyzed using the 2ΔΔ-Ct method. All primers are shown in Supplementary Table 1.

### Cytokine arrays

The expression of angiogenic cytokines in cell lysates was evaluated using a high-throughput Human Angiogenesis Array Kit (ARY007, R&D Systems, Minneapolis, MN, USA). Cell lysates were collected following co-culture with either exosomes or PBS for 24 hrs. Protein concentrations were estimated using the BCA method (Solaribo). A total of 200 μg protein was mixed with the biotinylated antibodies and incubated with the membrane overnight on a shaker at 4°C. The next day, the membrane was washed with 1x wash buffer for 10 min and then incubated with Streptavidin-HRP for 30 min. Following the incubation, the membrane was washed three times and developed using the ECL Reagent (Millipore, Billerica, MA, USA). Pixel density was quantified using the Quantity One software (Bio-Rad Version 4.6.2).

### ELISA

ANG-1 and ANG-2 concentrations in serum and hematoma samples were measured using an ELISA kit (R&D Systems) according to the manufacturer’s protocol.

### Immunocytochemistry

Purified exosomes were labeled with the PKH26 using the PKH26 Red Fluorescent Labeling Kit (Sigma-Aldrich, Cat. MIDI26, USA) according to the manufacturer’s protocol. PKH26-labeled exosomes were co-cultured with HUVECs at 37°C for 24 hrs. The cells were then stained with PKH67 (Sigma-Aldrich, Cat. MIDI67, USA) and DAPI (GeneCopoeia, Rockville, MD, USA). Imaging was performed using an Olympus IX71 microscope.

### *In vitro* tube formation assays

Matrigel (Sigma-Aldrich, USA) was thawed overnight at 4°C prior to use in all assays. A total of 50 μL of chilled Matrigel was added to each well of an ice-cold 96-well plate. The plates were then incubated for 60 min at 37°C to allow the Matrigel to solidify. Following gel formation, 10^4^ HUVECs were dissolved in 100 μL EGM-2 medium, seeded into each well, and then incubated for 6 hrs. Tube formation was analyzed using an IX81 inverted phase-contrast microscope (Olympus).

### *In vitro* vascular permeability assays

CultreCoat® 96 Well *In Vitro* Vascular Permeability Assays (Trevigen, Cat. 3475-096-K) were performed according to the manufacturer’s instructions. The collagen I-coated upper chambers were rehydrated with 50 μL of complete culture medium for 2 hrs at 37°C in a CO_2_ incubator. Following the incubation, 10^5^ cells were resuspended in complete culture medium, seeded into the upper chambers, and incubated for 72 hrs at 37°C. A total of 50 μL of FITC-Dextran was then added to the top chambers and the cells incubated for 5 min. Finally, the top chamber was removed and the absorbance of FITC-Dextran (485 nm excitation, 520 nm emission) measured.

### Rat model of acute SDH

The acute SDH rat model was established as described previously [40]. Briefly, rats were anesthetized with 10% chloral hydrate and then positioned in a stereotaxic frame (Stoelting, Wood Dale, IL). A small burr hole (0.9 mm diameter) was made using a sphenoid drill and the dura of the rat scuffed with a small hooked needle (0.3 mm diameter) under a microscope. A total of 300 μL autologous venous blood was collected from the angular vein, mixed with 100 μg of exosomes, and dissolved in 100 μL sterile PBS. The mixture was then injected into the subdural space at a rate of 50 μL/min using a 20-gauge Venflon catheter (BD Venflon, Helsingborg, Sweden).

### MRI

MRI was performed within 2 hrs of SDH induction to confirm the model was established successfully and measure lesion volume. Rats were fixed on the coil (3 T, CG-MUC19-H300-AG, Shanghai Chengguang Technology Co. Ltd.) and positioned in the MRI (GE3.0 T). Initial coronal T2-weighted images along the coronal view of the head were acquired without contrast injection. The slice thickness was 1 mm. SDH rats were either included or excluded from further analysis based on the initial set of MR images. Excluded rats demonstrated induction failure, cortex contusion or laceration, or severe edema surrounding the hematoma. All rats included in further analyses demonstrated hypointense hematomas and areas of cortical ischemia.

### Analysis of cognitive deficits using mNSS

Cognitive deficits were analyzed using the mNSS as described previously [41]. Tests were performed by two observers who were both blinded to the experiment 1, 3, 7, 14, and 21 days after establishment of the SDH model.

### Overexpression of miR-144-5p

MiR-144-5p overexpression was induced by transfecting miR-144-5p mimics (sense: 5′-GGAUAUCAUCAUAUACUGUAAG-3′; antisense: 5′-UACAGUAUAUGAUGAUAUCCUU-3′) with the siRNA Mate Kit (GenePharma, Cat. G04003, China). The following siRNA (sense: 5′-UUCUCCGAACGUGUCACGUTT-3′, antisense: 5′-ACGUGACACGUUCGGAGAATT-3′) was used as a negative control. Cells and hematoma membranes were harvested after 48 hrs. MiR-144-5p overexpression was confirmed by RT-PCR.

### Statistical analysis

All data were analyzed using SPSS V21.0. One-way analysis of variance (ANOVA) with least significant difference post hoc or unpaired T tests were used for all analyses. All values are expressed as the mean ± standard error of the mean (SEM). A p < 0.05 was considered statistically significant.

### Limitations

How the exosomes in the hematoma were generated were not studied in this study, which should be investigated in the further research.

## Supplementary Material

Supplementary Figures

Supplementary Tables 1 and 2

Supplementary Table 3

Supplementary Table 4

## References

[1] Henaux PL, Le Reste PJ, Laviolle B, Morandi X. Steroids in chronic subdural hematomas (SUCRE trial): study protocol for a randomized controlled trial. Trials. 2017; 18:252. 10.1186/s13063-017-1990-8 28583162 PMC5460366

[2] Jiang R, Zhao S, Wang R, Feng H, Zhang J, Li X, Mao Y, Yuan X, Fei Z, Zhao Y, Yu X, Poon WS, Zhu X, et al. Safety and Efficacy of Atorvastatin for Chronic Subdural Hematoma in Chinese Patients: A Randomized ClinicalTrial. JAMA Neurol. 2018; 75:1338–46. 10.1001/jamaneurol.2018.2030 30073290 PMC6248109

[3] Rauhala M, Luoto TM, Huhtala H, Iverson GL, Niskakangas T, Öhman J, Helén P. The incidence of chronic subdural hematomas from 1990 to 2015 in a defined Finnish population. J Neurosurg. 2019; 1–11. [Epub ahead of print]. 10.3171/2018.12.JNS183035 30901751

[4] Balser D, Farooq S, Mehmood T, Reyes M, Samadani U. Actual and projected incidence rates for chronic subdural hematomas in United States Veterans Administration and civilian populations. J Neurosurg. 2015; 123:1209–15. 10.3171/2014.9.JNS141550 25794342 PMC4575892

[5] You W, Zhu Y, Wang Y, Liu W, Wang H, Wen L, Yang X. Prevalence of and risk factors for recurrence of chronic subdural hematoma. Acta Neurochir (Wien). 2018; 160:893–99. 10.1007/s00701-018-3513-0 29532258

[6] Miranda LB, Braxton E, Hobbs J, Quigley MR. Chronic subdural hematoma in the elderly: not a benign disease. J Neurosurg. 2011; 114:72–76. 10.3171/2010.8.JNS10298 20868215

[7] Rohde V, Graf G, Hassler W. Complications of burr-hole craniostomy and closed-system drainage for chronic subdural hematomas: a retrospective analysis of 376 patients. Neurosurg Rev. 2002; 25:89–94. 10.1007/s101430100182 11954771

[8] Thotakura AK, Marabathina NR. The Role of Medical Treatment in Chronic Subdural Hematoma. Asian J Neurosurg. 2018; 13:976–83. 10.4103/ajns.AJNS_13_17 30459852 PMC6208261

[9] Holl DC, Volovici V, Dirven CMF, Peul WC, van Kooten F, Jellema K, van der Gaag NA, Miah IP, Kho KH, den Hertog HM, Lingsma HF, Dammers R; Dutch Chronic Subdural Hematoma Research Group (DSHR). Pathophysiology and Nonsurgical Treatment of Chronic Subdural Hematoma: From Past to Present to Future. World Neurosurg. 2018; 116:402–411.e2. 10.1016/j.wneu.2018.05.037 29772364

[10] Hua C, Zhao G, Feng Y, Yuan H, Song H, Bie L. Role of Matrix Metalloproteinase-2, Matrix Metalloproteinase-9, and Vascular Endothelial Growth Factor in the Development of Chronic Subdural Hematoma. J Neurotrauma. 2016; 33:65–70. 10.1089/neu.2014.3724 25646653 PMC4700393

[11] Sato S, Suzuki J. Ultrastructural observations of the capsule of chronic subdural hematoma in various clinical stages. J Neurosurg. 1975; 43:569–78. 10.3171/jns.1975.43.5.0569 1181389

[12] Stanisic M, Aasen AO, Pripp AH, Lindegaard KF, Ramm-Pettersen J, Lyngstadaas SP, Ivanovic J, Konglund A, Ilstad E, Sandell T, Ellingsen O, Sæhle T. Local and systemic pro-inflammatory and anti-inflammatory cytokine patterns in patients with chronic subdural hematoma: a prospective study. Inflamm Res. 2012; 61:845–52. 10.1007/s00011-012-0476-0 22527446

[13] Suzuki M, Endo S, Inada K, Kudo A, Kitakami A, Kuroda K, Ogawa A. Inflammatory cytokines locally elevated in chronic subdural haematoma. Acta Neurochir (Wien). 1998; 140:51–55. 10.1007/s007010050057 9522908

[14] Nanko N, Tanikawa M, Mase M, Fujita M, Tateyama H, Miyati T, Yamada K. Involvement of hypoxia-inducible factor-1alpha and vascular endothelial growth factor in the mechanism of development of chronic subdural hematoma. Neurol Med Chir (Tokyo). 2009; 49:379–85. 10.2176/nmc.49.379 19779281

[15] Hohenstein A, Erber R, Schilling L, Weigel R. Increased mRNA expression of VEGF within the hematoma and imbalance of angiopoietin-1 and -2 mRNA within the neomembranes of chronic subdural hematoma. J Neurotrauma. 2005; 22:518–28. 10.1089/neu.2005.22.518 15892598

[16] Zhang ZG, Buller B, Chopp M. Exosomes - beyond stem cells for restorative therapy in stroke and neurological injury. Nat Rev Neurol. 2019; 15:193–203. 10.1038/s41582-018-0126-4 30700824

[17] Bátiz LF, Castro MA, Burgos PV, Velásquez ZD, Muñoz RI, Lafourcade CA, Troncoso-Escudero P, Wyneken U. Exosomes as Novel Regulators of Adult Neurogenic Niches. Front Cell Neurosci. 2016; 9:501. 10.3389/fncel.2015.00501 26834560 PMC4717294

[18] Song L, Peng L, Hua S, Li X, Ma L, Jie J, Chen D, Wang Y, Li D. miR-144-5p Enhances the Radiosensitivity of Non-Small-Cell Lung Cancer Cells via Targeting ATF2. Biomed Res Int. 2018; 2018:5109497. 10.1155/2018/5109497 29850528 PMC5925000

[19] Zeng Z, Li Y, Pan Y, Lan X, Song F, Sun J, Zhou K, Liu X, Ren X, Wang F, Hu J, Zhu X, Yang W, et al. Cancer-derived exosomal miR-25-3p promotes pre-metastatic niche formation by inducing vascular permeability and angiogenesis. Nat Commun. 2018; 9:5395. 10.1038/s41467-018-07810-w 30568162 PMC6300604

[20] Gong M, Yu B, Wang J, Wang Y, Liu M, Paul C, Millard RW, Xiao DS, Ashraf M, Xu M. Mesenchymal stem cells release exosomes that transfer miRNAs to endothelial cells and promote angiogenesis. Oncotarget. 2017; 8:45200–12. 10.18632/oncotarget.16778 28423355 PMC5542178

[21] Karnati HK, Garcia JH, Tweedie D, Becker RE, Kapogiannis D, Greig NH. Neuronal Enriched Extracellular Vesicle Proteins as Biomarkers for Traumatic Brain Injury. J Neurotrauma. 2019; 36:975–87. 10.1089/neu.2018.5898 30039737 PMC6444902

[22] Otero-Ortega L, Laso-García F, Gómez-de Frutos M, Fuentes B, Diekhorst L, Díez-Tejedor E, Gutiérrez-Fernández M. Role of Exosomes as a Treatment and Potential Biomarker for Stroke. Transl Stroke Res. 2019; 10:241–49. 10.1007/s12975-018-0654-7 30105420

[23] Yang TT, Liu CG, Gao SC, Zhang Y, Wang PC. The Serum Exosome Derived MicroRNA-135a, -193b, and -384 Were Potential Alzheimer’s Disease Biomarkers. Biomed Environ Sci. 2018; 31:87–96. 29606187 10.3967/bes2018.011

[24] Heula AL, Ohlmeier S, Sajanti J, Majamaa K. Characterization of chronic subdural hematoma fluid proteome. Neurosurgery. 2013; 73:317–31. 10.1227/01.neu.0000430323.24623.de 23632762

[25] Wang D, Jiang R, Liu L, Dong JF, Zhang JN. Membrane neovascularization and drainage of subdural hematoma in a rat model. J Neurotrauma. 2010; 27:1489–98. 10.1089/neu.2009.1057 20486809

[26] Song H, Li X, Zhao Z, Qian J, Wang Y, Cui J, Weng W, Cao L, Chen X, Hu Y, Su J. Reversal of Osteoporotic Activity by Endothelial Cell-Secreted Bone Targeting and Biocompatible Exosomes. Nano Lett. 2019; 19:3040–48. 10.1021/acs.nanolett.9b00287 30968694

[27] Fiorella D, Arthur AS. Middle meningeal artery embolization for the management of chronic subdural hematoma. J Neurointerv Surg. 2019; 11:912–15. 10.1136/neurintsurg-2019-014730 30798265

[28] Moskala M, Goscinski I, Kaluza J, Polak J, Krupa M, Adamek D, Pitynski K, Miodonski AJ. Morphological aspects of the traumatic chronic subdural hematoma capsule: SEM studies. Microsc Microanal. 2007; 13:211–19. 10.1017/S1431927607070286 17490504

[29] Ito H, Yamamoto S, Saito K, Ikeda K, Hisada K. Quantitative estimation of hemorrhage in chronic subdural hematoma using the 51Cr erythrocyte labeling method. J Neurosurg. 1987; 66:862–64. 10.3171/jns.1987.66.6.0862 3572516

[30] Gao Z, Liu R, Liao J, Yang M, Pan E, Yin L, Pu Y. Possible tumor suppressive role of the miR-144/451 cluster in esophageal carcinoma as determined by principal component regression analysis. Mol Med Rep. 2016; 14:3805–13. 10.3892/mmr.2016.5691 27572636

[31] Li CY, Liang GY, Yao WZ, Sui J, Shen X, Zhang YQ, Peng H, Hong WW, Ye YC, Zhang ZY, Zhang WH, Yin LH, Pu YP. Identification and functional characterization of microRNAs reveal a potential role in gastric cancer progression. Clin Transl Oncol. 2017; 19:162–72. 10.1007/s12094-016-1516-y 27173517

[32] Chang CW, Wu HC, Terry MB, Santella RM. microRNA Expression in Prospectively Collected Blood as a Potential Biomarker of Breast Cancer Risk in the BCFR. Anticancer Res. 2015; 35:3969–77. 26124344 PMC4776637

[33] Li J, Wang R, Ge Y, Chen D, Wu B, Fang F. Assessment of microRNA-144-5p and its putative targets in inflamed gingiva from chronic periodontitis patients. J Periodontal Res. 2019; 54:266–77. 10.1111/jre.12627 30450635

[34] Yamada Y, Arai T, Kojima S, Sugawara S, Kato M, Okato A, Yamazaki K, Naya Y, Ichikawa T, Seki N. Regulation of antitumor miR-144-5p targets oncogenes: direct regulation of syndecan-3 and its clinical significance. Cancer Sci. 2018; 109:2919–36. 10.1111/cas.13722 29968393 PMC6125479

[35] De Rossi G, Whiteford JR. A novel role for syndecan-3 in angiogenesis. F1000 Res. 2013; 2:270. 10.12688/f1000research.2-270.v1 24555114 PMC3886797

[36] Elbaz A, Poirier O, Canaple S, Chédru F, Cambien F, Amarenco P. The association between the Val34Leu polymorphism in the factor XIII gene and brain infarction. Blood. 2000; 95:586–91. 10.1182/blood.V95.2.586 10627467

[37] Jones N, Iljin K, Dumont DJ, Alitalo K. Tie receptors: new modulators of angiogenic and lymphangiogenic responses. Nat Rev Mol Cell Biol. 2001; 2:257–67. 10.1038/35067005 11283723

[38] Fagiani E, Christofori G. Angiopoietins in angiogenesis. Cancer Lett. 2013; 328:18–26. 10.1016/j.canlet.2012.08.018 22922303

[39] Marqués-García F, Isidoro-García M. Protocols for Exosome Isolation and RNA Profiling. Methods Mol Biol. 2016; 1434:153–67. 10.1007/978-1-4939-3652-6_11 27300537

[40] Li T, Wang D, Tian Y, Yu H, Wang Y, Quan W, Cui W, Zhou L, Chen J, Jiang R, Zhang J. Effects of atorvastatin on the inflammation regulation and elimination of subdural hematoma in rats. J Neurol Sci. 2014; 341:88–96. 10.1016/j.jns.2014.04.009 24774750

[41] Gao C, Qian Y, Huang J, Wang D, Su W, Wang P, Guo L, Quan W, An S, Zhang J, Jiang R. A Three-Day Consecutive Fingolimod Administration Improves Neurological Functions and Modulates Multiple Immune Responses of CCI Mice. Mol Neurobiol. 2017; 54:8348–60. 10.1007/s12035-016-0318-0 27924525

