# Supplementary material for: Hematoma-derived exosomes of chronic subdural hematoma promote abnormal angiogenesis and inhibit hematoma absorption through miR-144-5p
Source: Aging (Albany NY). 2019 Dec 16;11(24):12147–64. doi: 10.18632/aging.102550 (PMC6949077; doi:10.18632/aging.102550)
Supplement: Supplementary Figures [file aging-11-102550-s004..pdf]

SUPPLEMENTARY FIGURES

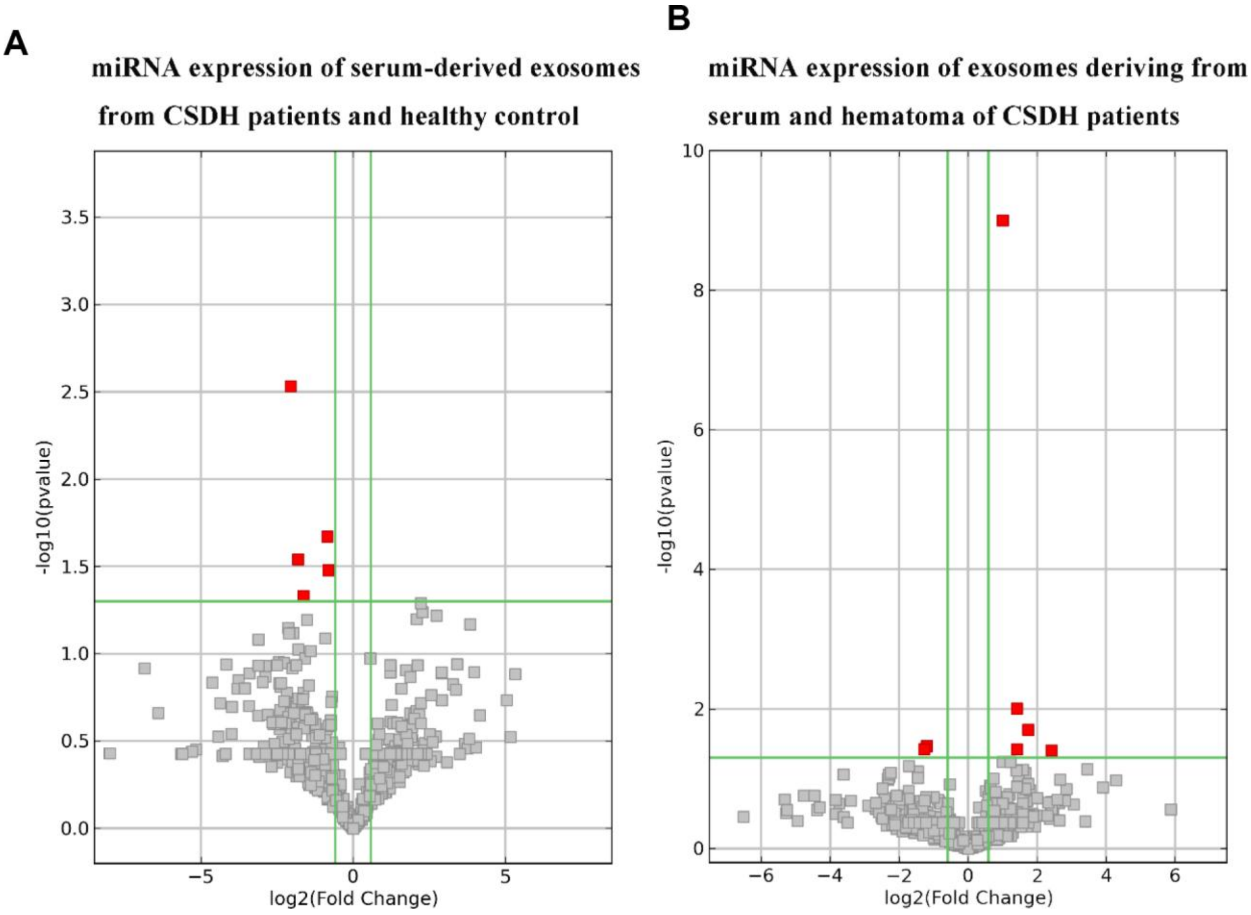

Supplementary Figure 1. Volcano plot of the miRNA sequencing data. (**A**) miRNA expression of serum-derived exosomes from CSDH patients and healthy control. (**B**) miRNA expression of exosomes deriving from serum and hematoma of CSDH patients.

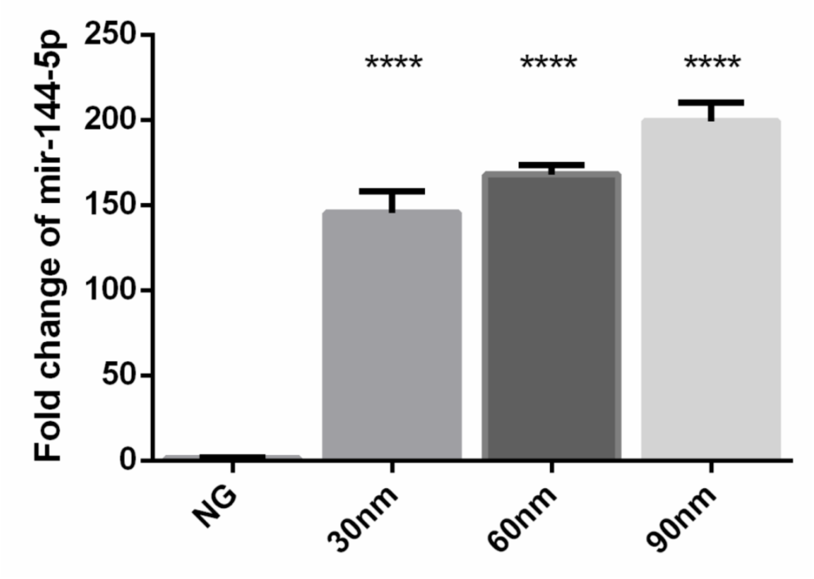

Supplementary Figure 2. The expression of mir-144-5p in HUVEC increased significantly after the transfection of mir-144-5p mimic than the negative control. ****p<0.0001 vs negative control.

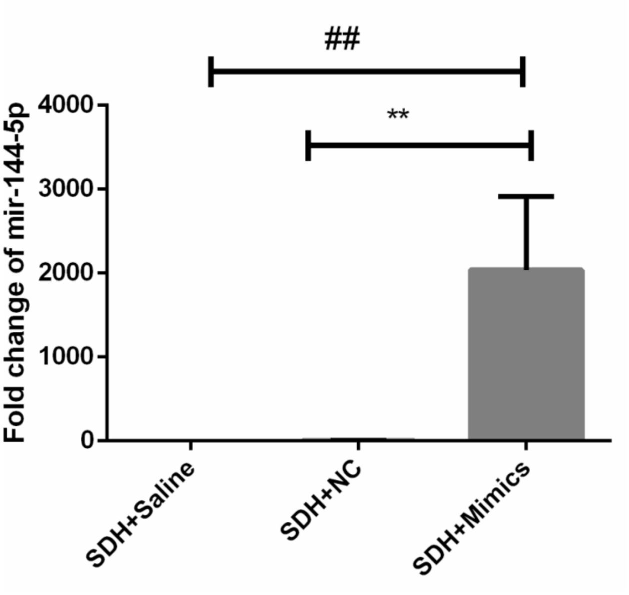

Supplementary Figure 3. The expression of mir-144-5p in the membrane of hematoma increased significantly after the transfection of mir-144-5p mimic than the negative control. **p<0.01, ##p<0.01.
