# Supplementary material for: Hematoma-derived exosomes of chronic subdural hematoma promote abnormal angiogenesis and inhibit hematoma absorption through miR-144-5p
Source: Aging (Albany NY). 2019 Dec 16;11(24):12147–64. doi: 10.18632/aging.102550 (PMC6949077; doi:10.18632/aging.102550)
Supplement: Supplementary Tables 1 and 2 [file aging-11-102550-s002..pdf]

SUPPLEMENTARY TABLES

Supplementary Table 1. Primers for RT-PCR.

Gene Name	Primer	Sequencing
Angpt2	1-Forward	AACATCCCAGTCCACCTGAG
	1-Reverse	GGTCTTGCTTTGGTCCGTTA
Angpt1	2-Forward	GGGGGAGGTTGGACTGTAAT
	2-Reverse	GAATAGGCTCGGTTCCCTTC
TEK	3-Forward	CAGCCCTGCTGATACCAAAT
	3-Reverse	GCGGTTTGTGACTTTCCATT
GAPDH	4-Forward	GGCCTCCAAGGAGTAAGACC
	4-Reverse	AGGGGAGATTCAGTGTGGTG
hsa-mir-144-5p	2-Forward	GGGGGATATCATCATATAC
	2-Reverse	CAGTGCGTGTCGTGGAGT
hsa-miR-16-5p(Control)	Forward	CAACGGAATCCCAAAAGCA
	Reverse	CAGTGCGTGTCGTGGAGT

Supplementary Table 2. Characterization and laboratory examination of healthy control and CSDH patients.

	Control	CSDH	p value
**Age, mean(SD)**	68.6(7.2)	68.5(6.9)	1.0
**Sex, male(%)**	7(70%)	15(75%)	0.5
**TP, mean(SD)**	63.4(3.7)	64.4(4.2)	0.5
**ALT, mean(SD),u/l**	18.1(13.8)	18.3(10.0)	1.0
**AST, mean(SD),u/l**	15.9(3.2)	16.2(3.5)	0.9
**Total cholesterol, mean(SD), mmol/l**	4.0(0.7)	3.6(0.8)	0.2
**Triglyceride, mean(SD), mmol/l**	1.1(0.4)	0.9(0.4)	0.4
**Erythrocyte (RBC),mean(SD),*10^{12} /l**	4.1(0.4)	4.2(0.5)	0.7
**Platelet (PLT),mean(SD), *10^{9} / l**	180.3(43.9)	198.3(74.1)	0.5
**Hemoglobin (HGB),mean(SD),g/l**	128.4(11.5)	130.2(15.5)	0.8
**Leukocyte (WBC), mean(SD),*10^{9} / l**	6.1(1.6)	6.9(2.3)	0.3
**Neutrophil, mean(SD),*10^{9} / l**	3.86(1.3)	4.7(1.9)	0.2
**Monocyte, mean(SD),*10^{9} / l**	0.47(0.12)	0.51(0.21)	0.7
**Lymphocyte, mean(SD),*10^{9} / l**	1.51(0.42)	1.48(0.41)	0.9
**Urea (BUN),mean(SD), mmol/l**	73.2(19.7)	72.3(14.0)	0.9
**Creatinine (Cr),mean(SD), umol/l**	5.6(1.3)	5.1(1.3)	0.3
**Glucose level, mean(SD), mmol/l**	5.58(1.23)	5.71(1.45)	0.8
